# The Surgical Management of Proximal Femoral Metastases: A Narrative Review

**DOI:** 10.3390/curroncol28050320

**Published:** 2021-09-28

**Authors:** Daniel Axelrod, Aaron M. Gazendam, Michelle Ghert

**Affiliations:** Division of Orthopaedic Surgery, Department of Surgery, McMaster University, Hamilton, ON L8N 4A6, Canada; daniel.axelrod@medportal.ca (D.A.); ghertm@mcmaster.ca (M.G.)

**Keywords:** metastatic bone disease, proximal femur metastases, endoprosthesis

## Abstract

The proximal femur is a common location for the development of bony metastatic disease. Metastatic bone disease in this location can cause debilitating pain, pathologic fractures, reduced quality of life, anemia or hypercalcemia. A thorough history, physical examination and preoperative investigations are required to ensure accurate diagnosis and prognosis. The goals of surgical management is to provide pain relief and return to function with a construct that provides stability to allow for immediate weightbearing. Current surgical treatment options include intramedullary nailing, hemiarthroplasty or total hip arthroplasty and endoprosthetic reconstructions. Oligometastatic renal cell carcinoma must be given special consideration as tumor resection and reconstruction has survival benefit. Both tumor and patient characteristics must be taken into account before deciding on the appropriate surgical intervention.

## 1. Introduction

As the burden of cancer is increasing, the number of patients living with metastatic cancer also continues to rise as systemic therapies lead to longer survival [1,2]. Patients with metastatic bone disease may live up to 4 years or longer, particularly those with breast and prostate carcinoma [3,4,5]. However, metastatic bone disease can cause debilitating pain, pathologic fractures, reduced quality of life, anemia or hypercalcemia [6].

Metastatic lesions in the proximal femur represent the most common location of metastases in the appendicular skeleton [7]. As a weight-bearing region, lesions in the proximal femur often present with functional pain or have a pathologic fracture [8]. Anatomically, roughly fifty percent of lesions are located in the femoral neck, while thirty percent are subtrochanteric and twenty percent are intertrochanteric [9]. 

## 2. Preoperative Assessment

A thorough history, physical examination and preoperative investigations are required in patients presenting with metastatic bone disease [10]. Although the majority of pathologic bony lesions in patients older than 40 are from metastatic bone disease, multiple myeloma and lymphoma are also common. The five most common primary malignancy sites to metastasize to bone are lung, kidney, breast, thyroid, and prostate cancer [7]. 

Skeletal metastases are the first clinical manifestation of malignancies in up to 25–30% of cases [11]. In patients presenting with a bone lesion of unknown origin, particular in the setting of a solitary lesion, a full preoperative workup must be performed in attempts to isolate a primary malignancy [10]. The workup for an unknown primary lesion includes blood work, imaging and biopsies of the lesion (Table 1). 

The treating physician should correlate the clinical history with the presentation of disease to ensure that a primary malignancy of bone is not missed and incorrectly assumed to be a metastatic deposit. A solitary bone lesion must be considered a primary bone tumour until proven otherwise [12]. To reduce the amount of operative interventions, a frozen section biopsy can be taken at the beginning of an operation to confirm that the lesion not a primary bone tumor, and then definitive fixation can be undertaken [13,14]. In the circumstances when the lesion is not carcinoma, or a primary bone tumour cannot be ruled out, the wound should be closed and fixation or prophylactic stabilization should be delayed until a definitive diagnosis is reached [14].

**Figure 1:**
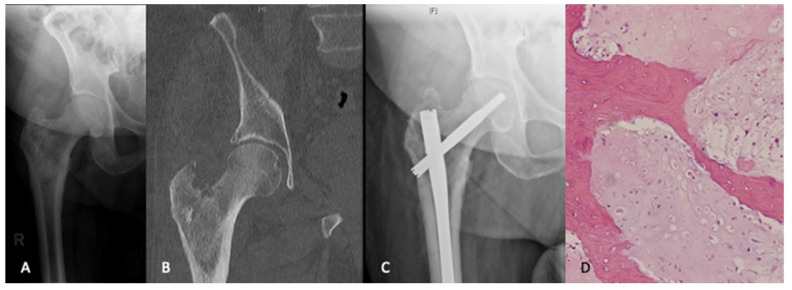
An 80-year-old male presented with ongoing right hip pain and lesion in the peritrochanteric region that was thought to be metastatic disease (**A**,**B**). A workup for an unknown primary lesion was negative. The patient underwent intramedullary nail fixation for impending pathologic fracture (**C**). The pathologic findings from intraoperative reamings were consistant with a diagnosis of chondrosarcoma (H&E, ×200). (**D**). Given the patients age and health status along with the morbidity of a curative procedure, the decision was made to pursue palliative care.

The “whoops” operation, management of a primary bone tumour with intramedullary nail fixation, is a disastrous event [15]. Intramedullary fixation contaminates the entire canal with tumour, as well as seeds the proximal and distal locking screw tracts, and the entry points for the nail in the proximal femur. The limb is often not salvageable, and a hip disarticulation may be required for local disease control (Figure 1). Patients with primary bone tumors who undergo inappropriate initial surgical management have higher rates of local recurrence and mortality, lower rates of limb salvage and worse functional outcomes [15,16,17]. 

## 3. Indications for Surgical Management

The management of metastatic bone disease is guided by the nature and location of the lesion, response to adjuvant therapy and the medical status and overall prognosis of the patient. Non-surgical treatment for metastatic bone disease, including radiotherapy, multimodal analgesia, hormonal therapies or bone modifying agents may be effective in certain circumstances [18]. 

Lesions that go on to pathologic fractures in the proximal femur require surgical fixation for pain control and mobilization [19]. Additionally, metastatic lesions at high risk of going on to eventual fracture should also be considered for prophylactic treatment. The best known prognostic tool to help inform us as to which lesions will go onto fracture is the Mirel’s criteria, which has good sensitivity to detect which lesions go on to fracture (88%) [20]. However, it has been shown in recent years to have limited inter-rater reliability and specificity (38%). More recently, multiple finite element and related analyses have been proposed to help prognosticate which patients with bone metastases will go onto fracture [21,22]. Sternheim et al. reported a sensitivity and specificity of their model of 100 and 68%, respectively, far better than the reported accuracy of Mirel’s score [23]. Prophylactic treatment before a fracture can improve patient quality of life, reduce pain scores and may reduce the mortality associated with the lesion. Recently, Phillip et al. showed that prophylactic fixation can reduce risk of death by up to 25% [24]. 

## 4. Prognosis

In addition to tumor factors, a patient’s overall prognosis and expected survival must play a role in treatment decisions. Several scoring systems, including the Eastern Cooperative Oncology Group (ECOG) and Karnofsky scores have been utilized to assess patient performance status and survival [25,26]. More recently, conventional prognostic models have been created to estimate survival based on patient specific characteristics [3]. For example, PATHFx is a tool that utilizes patient specific data to generate survival probabilities at varying time points and has been validated in various populations internationally [27]. Other models have attempted to predict survival more accurately using sophisticated machine learning technology [28]. For example, Thio et al. have developed a machine learning model which incorporates histology, metastases, previous therapy and a host of patient and laboratory factors. This is now freely available for open access use and has recently been validated [29]. Similarly, Sarahrudi et al. created a model which suggested that the median survival of patients who present with pathologic fractures of the proximal femur was 2.7 months, with an additional risk of death within one month if treatment including reconstruction with an endoprosthesis [30]. In addition to these models, certain clinical and laboratory findings have been found to be prognostic in this patient population [31]. Errani et al. demonstrated that pathologically elevated CRP (≥1.0 mg/dL) along with an unfavourable primary tumour diagnosis strongly predicted 12-month survival in this population [31].

## 5. Goals of Treatment

The primary goal of surgical management for patients with skeletal metastases is pain relief and the restoration (or preservation) of function [32]. Patients with metastatic lesions to the proximal femur may expect up to a 5-year survival, depending on the type of primary malignancy, its phenotype and patient factors [4,5]. Those with fractures due to metastatic bone disease will always heal more slowly than corresponding injury in non-pathologic bone [10]. Over than 50% of all pathologic fractures will never heal at time of final assessment, due to abnormal local biology and concomitant oncologic therapy [33]. Accordingly, any construct must offer sufficient support to the patient even if the native fracture does not go on to union. Consideration should be given for adjuvant treatment of metastatic lesions with radiation therapy, as advised by a multidisciplinary team including radiation and medical oncologists [34]. Radiotherapy should be strongly considered as an adjuvant treatment to reduce pain and potentially reduce the risk of local disease recurrence [34]. The literature is mixed regarding the ability of radiotherapy to reduce disease recurrence and may change in the future with the adoption of new systemic therapies that increase survival time [34,35,36].

The risks of perioperative complications, including deep venous thrombosis and pulmonary embolism, are higher amongst patients with metastatic disease when compared to those without [37]. Given this, it is critical that any pathologic fracture is treated with a construct that can allow for immediate weight bearing. This not only reduces the immediate risk of perioperative complications associated with immobility, but also improves the patient’s quality of life.

Preoperative imaging should be extensively scrutinized to evaluate the presence of other sites of disease. Full length films of the affected femur should be obtained to assess for more distant sites of metastases within the femur, while the acetabulum should be investigated for any concerning lesions [32]. If there are lesions along the length of the femur, a proximal femoral arthroplasty may not be sufficient and will pose a high risk of periprosthetic fracture. Similarly, a hemiarthroplasty in the presence of substantial acetabular disease burden may not alleviate the patient of their oncologic pain and may risk early catastrophic failure. All areas of the bone that are impacted by the disease should be addressed in any planned reconstruction [38].

There is substantial value in the utilization of angio-embolization for patients who present with highly vascular metastatic tumours. Embolization of metastatic bone lesions from renal cell cancer reduces units of blood transfusion after surgery by approximately one unit [39] and a recent systematic review found a low rate of embolization related complications [40]. Although the classic orthopedic teaching was to consider embolization of metastatic disease from thyroid cancer and multiple myeloma, this is not supported by the current literature.

Additionally, there is a subset of patients with solitary or oligometastatic disease in which resection is advantageous [25,41,42]. In oligometastatic disease, dissemination of tumour is limited to one or two distant sites, and the goal of treatment intent may be curative rather than palliative [41]. Patients with oligometastatic disease will likely have longer overall survival than those with disseminated metastases [42]. The decision to perform resection of metastatic deposits in a curative fashion will be dependent on patients anticipated survival benefit and disease prognosis, but aggressive management of patients with oligometastatic disease is likely to provide survival benefit in addition to providing symptom control [10]. In particular, retrospective studies have shown that isolated metastatic renal cell cancer deposits treated with en-bloc resection have been found to have increased overall survival when compared to intralesional techniques [25]. Ratasvuori et al. demonstrated that patients with renal cell carcinoma, en bloc resection of solitary metastases was associated with a fourfold longer survival than those treated with intralesional surgery [43]. In addition to the potential survival benefits, renal cell carcinoma is known to be resistant to adjuvant radiotherapy, which may increase the risk of tumor progression and potential hardware failure when treated with intralesional fixation [44,45]. Thus, the decision to perform en-bloc resection of oligometastatic disease with subsequent reconstruction, rather than intralesional treatment is dependent on tumour type and patients expected prognosis [43].

One should also consider quality of life (QoL) above and beyond the mortality benefit of treatment. Recent studies have attempted to measure the minimally clinical important differences (MCID) in treatment of patients with lower extremity metastases for multiple domains of QoL including PROMIS scores and the SF-36 score [46,47]. Bongers et al. report an MCID of 7.5 and 4.1 points for pain and physical function, respectively. This MCID sets a threshold for any treatment option for metastatic bone disease to exceed this before being considered. However, due to the limited literature available in this domain and the challenges in measuring QoL in this population, these MCID calculations should not be considered independantly, but rather in concert with the patients unique goals of care and their clinical picture when considering treatment decisions.

## 6. Surgical Management

### Osteosynthesis

In the proximal femur, lesions in the pertrochanteric and subtrochanteric region are often treated with an intramedullary nail (IMN). Intramedullary nails offer the benefit of being load sharing devices that allow for immediate weight bearing [48]. The majority of orthopedic surgeons will feel comfortable with this procedure and will have adequate instrumentation to perform this operation in most community settings. The contemporary literature demonstrates reasonably low complication rates and reliable improvements in pain and function in the majority of patients [49,50].

A variety of IMNs have been used historically, including standard antegrade IMN with fixation into the lesser trochanter. However, with no fixation into the femoral head or neck, these implants have fallen out of favour in the setting of pathologic fractures, given that the basicervical and neck regions are the most common sites of metastatic bone disease in the femur [45]. If future metastatic deposits are to develop, the patient will have no protection against a catastrophic future fracture. Thus, it is recommend that femoral lesions be treated with reconstruction style femoral nail with interlocking screws both proximally in the head and neck, as well as distally in the suprapatellar area [48]. In this same vein, it is recommended to utilize a long intramedullary nail to protect the entirety of the bone [10]. However, there is conflicting evidence in the literature as to whether this orthopaedic dogma is substantiated by evidence [51]. To increase rigidity of the construct, the maximum number of interlocking screws should be used. The use of cement is suggested as it provides immediate stability and has been shown to reduce revision rates [50].

A potential downside to the use of IMN is that they are fundamentally designed as a load-sharing device. In the setting if pathologic bone, there is often minimal healing and high rates of non-union, forcing the construct to act as a load-bearing device [52]. Over time, in patients with prolonged life expectancies, the loads placed on the implant can lead to hardware failure [53]. Increased survival has been demonstrated to be an independent predictor of hardware failure [54,55]. Similarly, patients with radioresistant tumors, such as renal carcinoma, are at risk of tumor progression and minimal bone healing. Patients with renal cell carcinoma have been shown to have increased rates of hardware failure after IMN [44,45]. It is imperative to consider both patient and tumor factors preoperatively to determine the appropriate implant choice.

Open reduction and internal fixation (ORIF) with plate and screw constructs, which are load-bearing, rather than load-sharing devices, are less favoured [56,57]. In distal femur metaphyseal/epiphyseal lesions or in younger patients with metaphyseal/epiphyseal lesions, fixed angle plates may be an appropriate option [32]. However, to use a plate, there must be sufficient bone stock such that the patient is allowed to weight bear immediately. The majority of the literature has demonstrated increased complication rates with ORIF when compared to IMN and reconstruction options in the proximal femur [57,58].

## 7. Reconstruction Options

In patients with isolated femoral head and neck lesions, proximal femoral resection and arthroplasty is preferred. Additionally, in patients with extensive lesions with substantial bone loss, or with tumours resistant to radiation therapy, proximal femoral reconstruction is preferred as they are at high risk of non-union or hardware failure with IMN fixation. In circumstances with failed internal fixation, prosthetic reconstruction can offer a salvage operation [44]. Although arthroplasty does not protect the entire length of the femur, Boden et al. have demonstrated that arthroplasty is a safe intervention with isolated proximal metastases, with low risk of later development of metastases distal to the prosthesis [59]. In a case–control study, patients who underwent proximal femoral replacement experienced similar pain scores and functional outcomes to a group with pertrochanteric lesions treated with intramedullary nailing [49].

### 7.1. Standard Prosthesis

Lesions of the head and neck without acetabular involvement can be treated with a hemiarthroplasty or total hip arthroplasty [49,60]. The implant must provide sufficient support for the inferomedial calcar. The calcar may have bone loss due to the site of fracture or secondary to metastatic bone lesions. A calcar replacing or calcar supporting type implant should be considered in these cases. If there are other sites of more distal disease, a long-stemmed prosthesis which bypasses these deposits is recommended to reduce the likelihood of a future periprosthetic fracture at a stress riser [61].

Both hemiarthroplasty and total hip arthroplasty (THA) are excellent options for patients with metastatic bone disease in the proximal femur [49]. Hemiarthroplasty is technically less challenging than total hip arthroplasty and can be reliably performed by community general orthopedic surgeons. Compared to THA, hemiarthroplasty is a shorter operation with less blood loss, and has a lower risk of postoperative dislocation [62]. However, hemiarthroplasty may be associated with acetabular erosion and wear from the metal component on the intact acetabular cartilage [60]. Thus, for some younger patients with longer life expectancy, total hip arthroplasty may be preferred. However, in a recent large cohort, hemiarthroplasty was not associated with a high risk of acetabular erosions requiring revision operations in a population of patients require treatment for lesions in the proximal femur [60]. There remains a lack of consensus between hemiarthroplasty and THA when managing metastatic disease of the femoral head and neck [63].

For lesions in the peritrochanteric area, either arthroplasty or internal fixation may be appropriate [64]. The majority of the literature demonstrates equivalent results between arthroplasty and IMN [49,64,65]. Meynard et al. examined 309 patients with proximal femur metastases and demonstrated no difference in functional outcomes, hardware failures and overall survival between techniques [49]. Both techniques are indicated in the contemporary management of proximal femur metastases and the choice should be based on surgeon, patient and tumor factors, and anatomic location within the proximal femur.

### 7.2. Endoprostheses

**Figure 2:**
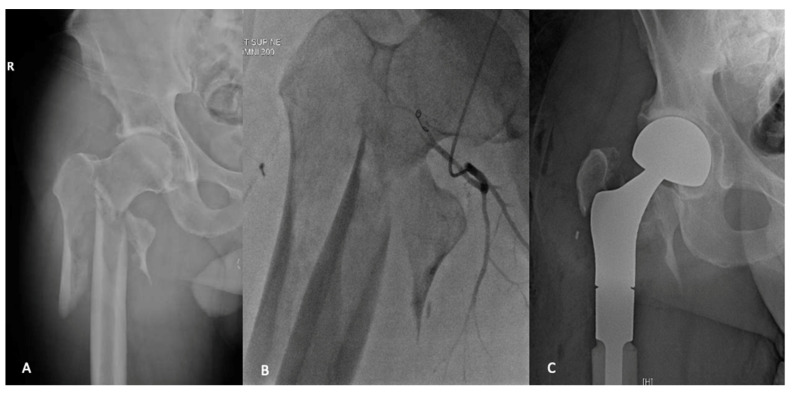
A 49-year-old male who presented with a pathologic fracture through an extensive lytic lesion in the pertrochanteric and subtrochanteric region (**A**). Staging and workup found this to be an isolated renal cell carcinoma metastases. Preoperative angio-embolization was undertaken (**B**), and the metastatic deposit was treated with en-bloc resection and reconstruction with proximal femoral replacement (**C**).

Endoprosthetic replacement may offer the best options for patients with extensive subtrochanteric bone loss. Endoprosthetic reconstructions offer a durable construct that allows for immediate weightbearing [66]. Several series have demonstrated that endoprosthetic reconstructions have improved implant survival when compared to IMN [57,66,67]. Harvey et al. evaluated 158 patients with proximal femur metastases extending into the subtrochanteric region [66]. They demonstrated similar functional outcomes and complications but improved implant longevity and reduced mechanical failure in the endoprosthetic group. The majority of orthopaedic oncologists recommend endoprosthetic reconstruction over IMN in patients with prolonged life expectancy [68]. A previous series of pathologic long bone fractures noted that the most important risk factor for implant failure was increased postoperative survival [55,69]. Endoprosthetic reconstructions also offer a reliable salvage operation in the setting of a failed fixation for pathologic fractures [44]. Finally, as mentioned previously, there is a growing movement that suggests that solitary lesions in patients with favourable histology and good overall prognosis may benefit from wide resection and reconstruction with an endoprosthesis (Figure 2) [70].

However, endoprosthetic reconstructions are associated with their own set of unique complications [56]. The intrinsic stability of a modular proximal femoral replacement is limited due to the lack of soft tissue attachments in the proximal femur. The dislocation rate after endoprosthetic reconstruction for metastatic disease remains high, ranging from 3% to 22% in the literature [8]. However, the vast majority of dislocations do not require revision surgery and can be treated with closed reduction alone [57]. Not surprisingly, infection rates have also been shown to be higher in endoprosthetic reconstructions when compared to IMN [56]. However, overall complication and revision rates are similar among different surgical strategies [56].

## 8. Conclusions

The proximal femur is a common location for the development of bony metastatic disease. Surgical management is aimed at providing pain control and immediate weightbearing to preserve function. Current treatment options include IMN, hemiarthroplasty or total hip arthroplasty and endoprosthetic reconstructions. Both tumor and patient characteristics must be taken into account before deciding on the appropriate surgical intervention.

## Figures and Tables

**Table 1:** Workup for unknown bone lesion in patients >40 years old.

Laboratory	Tests	Notes
	Complete Blood Count	
	Electrolyte panel	
	Bone biochemistry	
	Liver Function Tests	
	Thyroid function tests	
	Prostate specific antigen	
	Inflammatory Markers	
	Serum protein electrophoresis/Urine protein electrophoresis	
Imaging	Orthogonal radiographs	Full length of affected bone and contralateral side
	Whole body bone scan	
	CT chest, abdomen, pelvis	
	Cross-sectional imaging of involved extremity	MRI or CT depending on patient factors
Biopsy	Open or image-guided	Follow biopsy principles

## Data Availability

No new data was created or analyzed in this study. Data sharing is not applicable to this article.
